# A Multilevel Spatial Survival Analysis of Patients in Texas with End-Stage Renal Disease

**DOI:** 10.3390/healthcare13233028

**Published:** 2025-11-24

**Authors:** Dongeun Kim, Yongwan Chun, Daniel A. Griffith

**Affiliations:** School of Economic, Political and Policy Sciences, The University of Texas at Dallas, Richardson, TX 75080, USA; dagriffith@utdallas.edu

**Keywords:** survival analysis, multilevel spatial modeling, Moran eigenvector spatial filtering, renal disease, spatial autocorrelation

## Abstract

Background/Objectives: This study investigates end-stage renal disease cases in Texas using a multilevel spatial survival modeling framework. The objective is to evaluate a multilevel model specification that incorporates regional as well as individual factors, and that can be extended with random effects capturing unexplained variation in the independent variables; these random effects can be partitioned into simultaneous spatially structured and spatially unstructured components. Methods: The analysis uses data from 109,018 adult patients who initiated end-stage renal disease treatment between 2009 and 2018, obtained from the United States Renal Data System. This paper presents this model structure for survival analysis using Moran eigenvector spatial filtering, providing an alternative way to conduct advanced spatial survival analysis. Results: Clinical variables, particularly age, cardiovascular comorbidities, and transplant status, are dominant predictors of survival. Racial disparities are observable, with Asian and Black patients exhibiting lower mortality risk relative to White patients. Socioeconomic indicators (poverty, urbanicity, and unemployment rate) show attenuated significance after adjusting for spatial and aspatial random effects, indicating their impact is partly mediated through unobserved regional heterogeneity and spatial autocorrelation. Conclusions: These findings underscore the necessity of accounting for spatial dependencies and multilevel structures in survival analysis to avoid potentially biased inferences. The devised approach can offer a robust framework for guiding geographically targeted health interventions and resource allocation aimed at improving end-stage renal disease patient outcomes and reducing health disparities across diverse regions.

## 1. Introduction

End-stage renal disease (ESRD), the final stage of chronic kidney disease (CKD), occurs when kidneys irreversibly lose their filtering function, requiring dialysis or kidney transplantation. Patients with ESRD face elevated mortality risks due to a combination of clinical comorbidities and demographic and socioeconomic influences [1]. Although medical advances have improved individual outcomes, disparities in survival rates persist across geographic regions and population groups, underscoring the need to examine contextual factors alongside clinical ones.

Traditional survival analysis methods, such as the Cox proportional hazards (CPH) model, provide important insights into individual-level predictors of survival while often failing to incorporate regional characteristics and spatial dependencies [2,3]. In demographically diverse states such as Texas, neglecting such spatial structures can lead to biased parameter estimates and overlooked existing patterns of vulnerability.

A growing body of research demonstrates that socioeconomic status (SES) is a critical determinant of health outcomes, including ESRD survival. Low income, limited education, lack of insurance, and residence in high-poverty areas contribute to delayed diagnosis, underutilization of preventive services, and reduced access to transplantation [4,5,6]. SES disparities are further compounded by race and ethnicity. Minority groups, particularly African American and Hispanic populations, are disproportionately represented in lower SES strata, and hence often face systemic barriers to adequate nephrology care, timely dialysis initiation, and transplantation [7,8]. These inequities reflect deeply entrenched structural challenges in the United States (U.S.) healthcare system and are especially salient in Texas.

Spatial factors also shape ESRD outcomes. Geographic accessibility to care, neighborhood deprivation, and regional healthcare infrastructure influence disease progression and survival. Studies across cancer, cardiovascular disease, and kidney disease confirm that geographic disparities in healthcare access produce systematic survival differences between urban and rural communities [9,10,11]. For ESRD specifically, clusters of late dialysis initiation and high mortality have been identified in socioeconomically disadvantaged and racially segregated regions, highlighting the role of spatial context in driving disparities.

To address these complexities, multilevel survival models have been used to capture both individual- and group-level effects [12,13,14]. These models allow unobserved heterogeneity across geographic units to be represented through random effects, reducing omitted variable bias and improving inference [15]. More recently, Moran eigenvector spatial filtering (MESF) has been applied in health research to capture latent spatial autocorrelation within a frequentist framework [16,17].

Beyond Bayesian spatial survival models, recent non-Bayesian approaches have incorporated space via shared spatial random effects in Cox-type models, demonstrating feasibility for disease mortality analyses and offering a practical comparator to MESF [18]. In parallel, the spatial epidemiology literature emphasizes diagnostics such as Moran’s *I* for global dependence and local indicators of spatial association (LISA) for localized clustering—tools that motivate filtering latent spatial signal in regression frameworks [19]. Reviews in chronic disease epidemiology likewise stress mapping, geographic correlation, and clustering as core spatial lenses with direct policy relevance, underscoring the value of models that clarify spatial structure while retaining interpretable covariate effects [19]. Finally, machine learning survival methods (e.g., random survival forests; deep Cox–type networks) provide flexible prediction benchmarks but serve different aims than MESF’s explicit control of spatial autocorrelation [20].

While Bayesian spatial survival models are highly flexible and permit the use of informative priors, they are computationally intensive and sensitive to prior choices. In contrast, MESF provides a more practical alternative in the context of frequentism. By introducing eigenvectors that capture latent spatial processes in the model, MESF accounts for spatial autocorrelation without requiring estimation of a full spatial covariance structure. Although MESF can also be implemented within a Bayesian framework, its frequentist formulation offers a simpler and computationally efficient approach that can be readily estimated using standard tools available in common statistical software such as R 4.5.2, SAS 9.4M9, Stata 19, and SPSS 31.

Building on these advances, this study applies a multilevel spatial survival framework to ESRD patients in Texas. By integrating individual-level clinical and demographic characteristics with county-level socioeconomic indicators, and by combining random effects with MESF, we seek to produce more accurate and geographically sensitive survival estimates. This approach enables the decomposition of mortality risk into spatially structured and unstructured components, generating insights that can inform targeted interventions and equitable resource allocation for vulnerable populations in Texas and beyond.

## 2. Case Study Landscape and Data Sources

**Figure 1:**
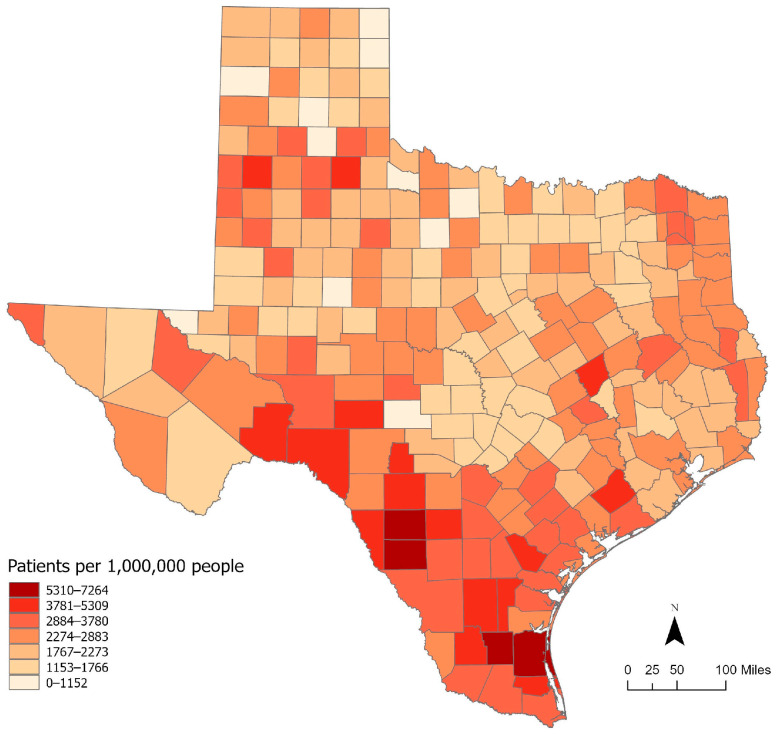
ESRD patients per 1,000,000 people in Texas between 2009 and 2018.

The study area is the state of Texas, a geographically expansive and demographically diverse region that offers an ideal geographic context for examining spatial and hierarchical disparities in health outcomes among patients with ESRD (Figure 1). Leveraging a large, geocoded dataset, the analysis for this paper investigates both individual- and county-level determinants of survival, capturing variation across urban and rural areas as well as among populations with differing socioeconomic/demographic characteristics.

The patient data were obtained from the United States Renal Data System (USRDS), maintained by the National Institute of Diabetes and Digestive and Kidney Diseases [21]. This dataset offers geocoded ESRD patient records aggregated to the zip code level, enabling spatially detailed assessments of treatment patterns and survival outcomes across Texas. This paper analyzes a total of 109,018 adult patients (aged 18 or older) who began ESRD treatment between 1 January 2009 and 31 December 2018. These data provide detailed clinical and demographic attributes necessary for a robust survival analysis at the patient level. To contextualize individual outcomes within broader geographic patterns, county-level socioeconomic/demographic data were integrated to examine the influence of structural conditions on mortality risk. Complementary contextual data were obtained from the U.S. Census Bureau via data.census.gov, including variables such as urbanicity rate, percent married, high school graduation rate, poverty rate, disability rate, veteran population rate, and unemployment rate. These socioeconomic indicators provide critical insight into the external factors that shape healthcare access and outcomes for ESRD patients. By integrating these data with both individual and aggregate geographic scales, this study expects to uncover spatial dimensions of health disparities in addition to patient-level risk factors. Another anticipation is that it can contribute to the development of targeted, data-driven interventions aimed at improving survival among vulnerable ESRD populations in Texas.

## 3. Methodology

This study employs a multilevel spatial survival modeling approach to comprehensively evaluate the clinical, socioeconomic/demographic, and contextual factors associated with mortality among patients diagnosed with ESRD in Texas. In order to accommodate individual patient characteristics and geographic contextual covariates simultaneously, the methodological framework utilizes a combination of the CPH model with hierarchical random effects modeling. Furthermore, this paper presents a decomposition of random effects into their spatially structured (SSRE) and unstructured (SURE) components using MESF. This integrative design was crafted to address unobserved heterogeneity at the regional level while explicitly accounting for spatial autocorrelation in addition to independent variables, which can result in enhanced results. The dependent variable in this study is survival time, defined as the number of months from the date of ESRD diagnosis to death. Censoring occurs when patients were either alive at the end of a follow-up (31 December 2018) or had incomplete follow-up information. Among the 109,018 adult ESRD patients included, approximately 54.52% were censored cases. Although the CPH model assumes censoring to be non-informative, certain mechanisms such as transplantation or withdrawal from dialysis may introduce informative censoring. To mitigate this potential bias, transplant status was incorporated as a covariate. Predictor variables were organized into three domains: clinical, demographic, and contextual socioeconomic.

Clinical variables for individual patients were obtained from the USRDS. One such variable is age at first ESRD service, originally recorded in 5-year intervals but reclassified into four clinically relevant categories based on the distribution of counts: 18–44 (reference category), 45–64, 65–74, and 75 years or older. Another key variable is transplant status, categorized into three groups: patients who have received no kidney transplant (reference category), those who have received one transplant, and those who have received two or more transplants. Cause of death is also included as a clinical variable, grouped into Cardiac, Vascular, and Other (reference category). Additionally, the primary disease causing renal failure is captured through categories such as Diabetes (reference category), Cystic Kidney, Glomerulonephritis, Hypertension, Other Urologic conditions, and Other. These variables provide critical insights into individual-level disease severity, treatment history, and clinical complexity influencing patient survival.

Demographic characteristics were also measured at the patient level, specifically gender and race [22,23,24]. Gender is operationalized as a binary variable indicating whether the patient is male or not male. Race is classified according to standard U.S. federal guidelines: White (reference category), Black or African American, American Indian or Alaska Native, Asian, and Other. Including these demographic variables enables a focused examination of disparities in survival outcomes across racial and sex-based subpopulations.

Socioeconomic and contextual indicators are derived from county-level data sourced from the U.S. Census Bureau’s American Community Survey. These county-level variables capture broad structural and environmental determinants of health, including urbanicity rate, percent married, high school graduation rate (as a proxy for education), poverty rate, disability rate, veteran population rate, and unemployment rate [5,25,26]. These contextual variables provide a critical framework for understanding how geographic differences in social and economic neighborhood conditions influence ESRD patient outcomes.

This paper summarizes and compares estimates for three nested models to assess the incremental value of incorporating random effects and spatial autocorrelation adjustments. One important rationale is that a survival analysis with geographic effects can be further enhanced with a model specification that accommodates both SSRE and SURE. The nested structure of these three models can present this enhancement by making comparisons with none, total random effects only, or their two decomposed components (SSRE and SURE). The modeling begins with the baseline CPH model, which provides a reference specification using only fixed effects. Next, it is extended to the model with county-level random intercepts to capture unobserved heterogeneity across geographic areas. These random effects are expected to have spatial and non-spatial components in a mixed way. A spatial eigenvector filter can capture spatial components from the mixed random effects. In the final model specification, the eigenvector spatial filter is introduced as an independent variable so that non-spatial random effects can be estimated. That is, this model can show a successful decomposition of the random effects term into its constituent SSRE and SURE components. This final model can not only improve the model fit but also furnish a way to explore geographic patterns that are unexplained by other covariates. These models are discussed in the following subsections.

### 3.1. Model 1: The CPH Model (Baseline)

The first and foundational model is the standard CPH model, which estimates the instantaneous hazard—or the risk of death—given patient-level and county-level covariates. The CPH model is widely used in biomedical research due to its flexibility in modeling survival data without requiring the specification of the baseline hazard function [27]. Formally, the hazard function can be expressed as the following equation:

(1) $$h\left(t|X\right)=h_{0}(t)\cdot exp(\beta_{1}x_{1}+\beta_{2}x_{2}+\ldots +\beta_{p}x_{p}),$$

where $h_{0}(t)$ denotes the unspecified baseline hazard function, $x_{1},\;x_{2},\;\ldots ,\;x_{p}$ represent the covariates included in the model, and $\beta_{1},\;\beta_{2},\;\ldots ,\;\beta_{p}$ are the respective regression coefficients. The hazard ratios obtained from this model quantify the relative changes in the hazard associated with each covariate, under the proportional hazards assumption—that is, the relative hazard remains constant over time.

### 3.2. Model 2: The CPH Model with County-Level Random Effects

Survival outcomes may be correlated within geographic units due to shared environmental exposures, healthcare access, and/or resource availability, among other common factors. Although county-level variables are included in this model, other relevant variables can still be missing. This omitted variables issue can be addressed with county-level random effects. This hierarchical adjustment modifies the hazard function to explicitly include random intercepts for each county as expressed by the following equation:

(2) $$h\left(t|X,r\right)=h_{0}\left(t\right)\cdot \exp\left(\beta_{1}x_{1}+\beta_{2}x_{2}+\ldots +\beta_{p}x_{p}+r\right),$$

where r represents county-specific random effects, which are assumed to be independently and identically distributed, following a normal distribution with mean zero and constant variance. This distributional assumption is standard in multilevel survival models and helps capture latent, non-spatial heterogeneity across counties. These random effects are expected to capture unobserved heterogeneity, particularly that described by missing variables. This modification allows the CPH hazard to vary across counties, thus appropriately accounting for hierarchical clustering in the data and reducing potential bias in the estimated effects of individual-level and contextual variables [28,29]. This random effects model is estimated with the *coxme* package in R.

### 3.3. Model 3: The Multilevel Spatial CPH Model with SSRE and SURE Terms

Spatial autocorrelation—where nearby geographic units exhibit more (dis)similar outcomes than those farther apart—can significantly bias traditional survival analyses if left unaddressed. The third model explicitly accounts for spatial dependencies using the MESF technique. This methodology is a robust spatial statistical technique that introduces prominent eigenvectors extracted from a doubly centered spatial weights matrix into a regression model as additional covariates, which in turn are expected to capture and control for latent spatial structures [16,30].

The MESF utilizes eigenvectors from a transformed spatial weights matrix C, say MCM, where $M=(I-{\mathrm{11}}^{T}/n)$, **I** is an n×n identity matrix, 1 is an n × 1 vector of ones, and n is the number of spatial units. The eigenvectors, E, via spectral decomposition (that is, $MCM=E\Lambda E^{T}$ where Λ is the diagonal matrix of their corresponding eigenvalues) are orthogonal, and each of them represents a distinct spatial pattern with an associated level of spatial autocorrelation. MESF introduces these eigenvectors as independent variables that function as spatial autocorrelation filters.

Here, an SSRE term is constructed solely from information latent in spatially autocorrelated regression residuals using a set of selected MESF eigenvectors to describe part of the random effects of Model 2, namely r. The MESF for the random effects can be expressed as

(3) $$r=E_{k}\beta_{E_{k}}+e$$

where $E_{k}$ represents a subset of k selected eigenvectors from the full set E, $\beta_{E_{k}}$ are its corresponding regression coefficients, and e denotes residuals with no meaningful spatial autocorrelation. These $E_{k}$ are expected to effectively filter out spatial autocorrelation from the county-level random effects. Here, the MESF is implemented with a binary spatial weights matrix based on continuity (i.e., shared boundaries) among counties. Prior research has shown that, as long as the chosen spatial weights matrix adequately represents the underlying spatial structure, results tend to be robust across alternative spatial weights matrix specifications [31,32]. Eigenvector choice is based on the significance of regression coefficients in a stepwise selection procedure.

The third model simultaneously integrates both county-level random effects and MESF-derived eigenvectors. That is, this extends Model 2 by introducing $E_{k}$ in Equation (3) as additional independent variables. This model can be expressed as follows:

(4) $$h\left(t|X,r\right)=h_{0}(t)\cdot exp(\beta_{1}x_{1}+\beta_{2}x_{2}+\ldots +\beta_{p}x_{p}+E_{k}\beta_{E_{k}}+r).$$

Hence, $E_{k}\beta_{E_{k}}$ denotes SSRE and r denotes SURE, modeled as county-level random intercepts that are assumed to follow a normal distribution with mean zero and constant variance. Because spatial autocorrelation is captured by SSRE, SURE is expected to account for non-spatial unobserved heterogeneity and is included under the standard assumption of normally distributed random effects. That is, this specification enables the incorporation of both SSRE and SURE in the traditional regression specification using standard tools when repeated measures (i.e., a space–time series) are available, which tend to be compatible with Bayesian specifications, even ones in which prior distributions substitute for multiple measurements. This model is expected to effectively separate spatial from non-spatial variance, which yields a robust result and helps understand spatial patterns in ESRD mortality.

The performances of the models are assessed with several diagnostic checks and model selection criteria. The proportional hazards assumption, foundational to the Cox model, is assessed using residual-based tests (e.g., Schoenfeld residuals). Model goodness-of-fit is compared using Akaike information criterion (AIC) values and log-likelihood statistics, with lower values indicating better model performance. These rigorous diagnostic procedures ensure that each model’s assumptions are validated and that the resulting hazard estimates are statistically sound and meaningful.

The integrative methodological approach outlined here provides a powerful analytical framework to disentangle complex relationships between individual-level clinical and demographic factors and county-level socioeconomic conditions. By explicitly accounting for spatial autocorrelation and regional heterogeneity (e.g., missing variables), this study accurately identifies regions and population subgroups with elevated ESRD mortality risk, guiding targeted public health interventions and resource allocation. Ultimately, the findings from this comprehensive methodological approach can inform evidence-based strategies aimed at reducing disparities and improving survival outcomes for ESRD patients throughout Texas.

## 4. Results

The performances of the three models are compared using AIC and log-likelihoods. Each successive model incorporates increasing additional spatial complexity, thereby enhancing the interpretability of spatial effects. Model 2, which introduces county-level random effects, shows a clear improvement over Model 1, with the log-likelihood increasing from −526,091 to −525,963) and a corresponding reduction in AIC (from 1,052,230 to 1,051,903). This improvement suggests that unobserved county-level factors, potentially linked to healthcare infrastructure, environmental exposures, or regional policy, account for a portion of the mortality risk. Model 3, which combines both spatially structured (SSRE) and unstructured (SURE) random components, provides the best overall performance, achieving the lowest AIC (1,051,873) and a log-likelihood of −525,911. Note that 19 eigenvectors are selected for the SSRE construction. The likelihood ratio tests in Table 1 confirm the model improvements with the inclusion of SSRE and SURE. Note that the models do not have multicollinearity issues, rendering low VIF values for the independent variables (1.09–1.89 without the eigenvectors, and 1.62–2.06 with the eigenvectors).

Table 2, Table 3 and Table 4 present the results of the three models. Across the three models, several individual-level clinical and demographic covariates consistently emerged as significant predictors of survival. Patients who received a transplant experienced markedly improved survival. Those who had one transplant had an HR of approximately 0.103 (95% CI: 0.096–0.112, *p* = 0.000), while patients with two or more transplants had an even lower HR of about 0.066 (95% CI: 0.035–0.123, *p* = 0.000). These results indicate that transplantation substantially reduces mortality risk. Cause of death was also highly significant: cardiac deaths (HR ≈ 2.11–2.14, *p* = 0.000) and vascular deaths (HR ≈ 2.30–2.31, *p* = 0.000) more than doubled the risk of mortality, underscoring the strong influence of comorbidity conditions. Primary disease was another influential factor. Compared with diabetic nephropathy, patients with cystic kidney disease (HR ≈ 0.60, *p* = 0.000) or glomerulonephritis (HR ≈ 0.81, *p* = 0.000) had improved survival, while those with other causes exhibited an increased risk (HR ≈ 1.26–1.28, *p* = 0.000). Patients diagnosed with other urologic causes also demonstrated better survival (HR ≈ 0.84, *p* = 0.000).

Age was one of the strongest predictors of survival. Relative to the reference group of patients under 45 years, those aged 45–64 had an HR of about 1.66 (95% CI: 1.595–1.723, *p* = 0.000), those aged 65–74 had an HR of approximately 2.63 (95% CI: 2.535–2.739, *p* = 0.000), and those aged 75 and older had an HR exceeding 4.19 (95% CI: 4.029–4.372, *p* = 0.000). These findings illustrate a steep increase in mortality risk with advancing age. By contrast, sex was not statistically significant in any of the models (HR ≈ 1.00, *p* = 0.55).

Racial differences were evident and consistent across all three specifications. Asian patients had an HR of about 0.74 (95% CI: 0.692–0.804, *p* = 0.000), while Black/African American patients had an HR of roughly 0.86 (95% CI: 0.842–0.888, *p* = 0.000), both indicating lower mortality relative to White patients. Patients identifying as “Other” also exhibited reduced mortality risk (HR ≈ 0.87, *p* = 0.000). In contrast, American Indian/Alaska Native patients did not differ significantly from White patients (*p* > 0.18). These results align with previous research documenting the so-called “survival paradox,” wherein minority patients on dialysis often experience better outcomes than their non-Hispanic White counterparts.

At the county level, socioeconomic and contextual factors displayed varying significance across the models. The following five variables are significant in Model 1: Urbanicity Rate (HR = 0.863, *p* = 0.000), Poverty Rate (HR = 0.989, *p* = 0.000), Disability Rate (HR = 1.009, *p* = 0.000), Veteran Population Rate (HR = 0.992, *p* = 0.000), and Unemployment Rate (HR = 1.008, *p* = 0.031). However, only two variables are significant in Models 2 and 3: Poverty Rate (HR = 0.993, *p* = 0.006) and Disability Rate (HR = 1.010, *p* = 0.013) in Model 2, and Disability Rate (HR = 1.009, *p* = 0.019) and Veteran Population Rate (HR = 1.011, *p* = 0.004) in Model 3. That is, when county-level random effects and/or spatial autocorrelation at the county level are accounted for, the inferential results are different. Given that Model 3 is preferred over the other two models, it is appropriate to interpret their coefficients based on Model 3.

Taken together, these results highlight that although some county-level contextual factors appear important in simpler specifications, their effects attenuate once spatial dependence and unobserved heterogeneity are explicitly modeled. The final specification indicates that county-level influences on ESRD survival are partly contextual as well as strongly structured by unobserved spatial processes.

### Spatial Pattern Diagnostics

**Figure 2:**
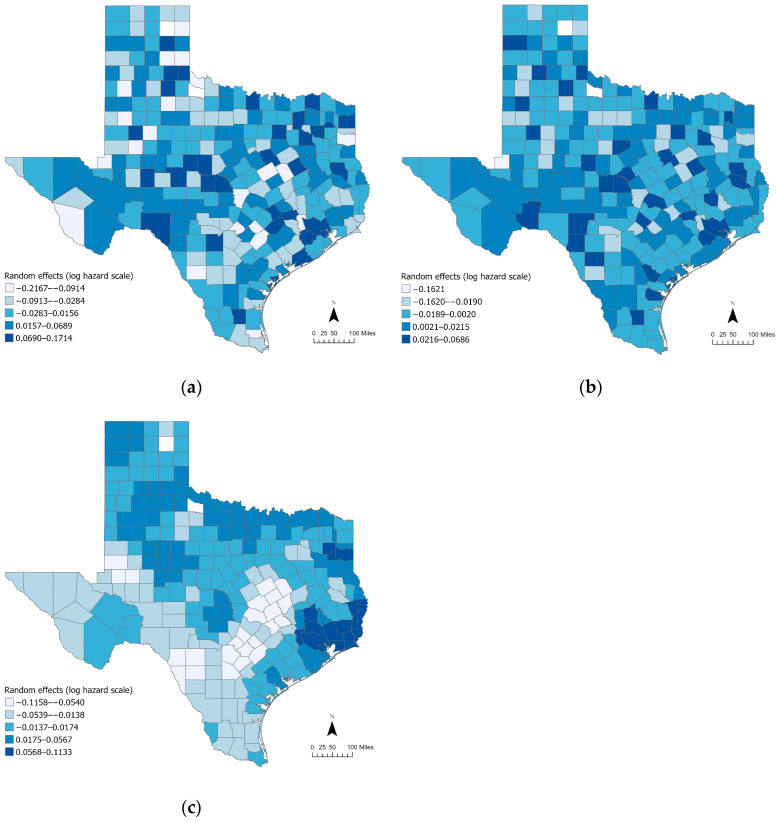
Spatial patterns of (**a**) Model 2 random effects (Moran’s *I* = 0.2547, *p* = 0.0000), (**b**) Model 3 SSRE (Moran’s *I* = 0.7960, *p* = 0.0000), (**c**) Model 3 SURE (Moran’s *I* = −0.0521, *p* = 0.9022).

To assess the spatial structure of unexplained mortality risk, the random effects from Models 2 and 3 were examined using Moran’s *I* statistic and choropleth maps. For Model 2, the random effects geographic distribution exhibits strong positive spatial autocorrelation, with a Moran’s *I* of 0.2547 (*p* = 0.0000). This weak-to-moderate spatial correlation indicates that counties with similar levels of mortality risk tend to be geographically clustered. Figure 2a illustrates distinct spatial patterns in the random effects. Elevated values are concentrated in the northern and central counties, particularly in the Panhandle and parts of east-central Texas, reflecting localized areas of heightened unexplained mortality risk. Conversely, lower random effects are observed across southern Texas and the western border regions. These patterns suggest that residual spatial variation persists after accounting for fixed effects, likely due to unobserved spatially clustered determinants of mortality.

Model 3 has the MESF terms to capture spatially structured variation while simultaneously estimating SURE. This decomposition allows the model to isolate two distinct components: SSRE and SURE, whose geographic distributions are shown in Figure 2b,c. The SSRE surface (Figure 2b) displays a smooth spatial gradient across the state, with high random effects (darker shading) concentrated in eastern and southeastern Texas and lower values toward the west, indicating persistent spatial structure after adjusting for covariates. The strongly structured nature of this component is confirmed by a Moran’s *I* value of 0.7960 (*p* = 0000), indicating strong positive spatial autocorrelation captured by the MESF terms.

Meanwhile, the SURE component (Figure 2c) shows no statistically meaningful spatial clustering (Moran’s *I* = −0.0521, *p* = 0.9022). Consistent with the map, higher and lower values occur as small, scattered pockets interspersed with neighboring counties of different magnitude rather than forming contiguous belts. This absence of global spatial autocorrelation indicates that the MESF terms in Model 3 absorbed the spatially structured dependence, leaving a spatially independent random effects that captures unexplained local heterogeneity.

Together, these sets of diagnostics demonstrate that spatial autocorrelation present in Model 2 is progressively reduced and ultimately decomposed through the use of MESF. The MESF-based model (Model 3) successfully disentangles structured and unstructured spatial variation, enabling the identification of both persistent spatial patterns (SSRE) and localized, non-spatial chance disparities (SURE) in county-level mortality risk. This approach provides an appealingly robust and interpretable framework for analyzing spatial health disparities.

## 5. Discussion and Conclusions

This study presents a multilevel spatial survival analysis model describing ESRD patients in Texas. This model shows how patient-level, county-level, and spatial structures collectively shape mortality outcomes. Its findings reinforce that survival among ESRD patients is not solely determined by medical or demographic factors but is deeply affected by geographic and socioeconomic conditions.

Model 3 displays the best model performance, demonstrating that a combination of county-level random effects and spatial filtering via MESF can accurately capture both spatially structured and unstructured heterogeneity, especially those affiliated with missing covariates. A model specification that does not appropriately accommodate these components—as in traditional survival formulations—can lead to biased estimates, misattributed effects, and weaker policy recommendations.

Clinical variables such as transplantation, age, and cardiovascular disease are the most influential predictors, which aligns with reports in the literature. These results underscore the importance of early intervention, transplant eligibility, and managing comorbidities. Consistent racial disparities—with Asian and Black patients showing better outcomes—invite further exploration into potential biological, cultural, and healthcare access-related mechanisms. Although Black and Asian patients demonstrate relatively better survival outcomes, this does not imply reduced vulnerability. At the policy level, this paradox should not be taken as diminished need but rather as a call to strengthen equity in transplantation access, post-dialysis care, and culturally tailored support. Such efforts can help ensure that apparent survival advantages do not mask underlying structural inequities.

To translate these patterns into action, the mapped risk surfaces, especially the SSRE component in Figure 2b, highlight persistent high-risk concentrations in eastern and southeastern Texas (e.g., Upper Gulf Coast/Houston periphery, Piney Woods, and adjacent coastal counties). For these counties, necessary concrete steps include expanding transplant referral and navigation, intensifying cardiovascular comorbidity management, increasing home/satellite dialysis capacity and tele-nephrology, as well as providing transportation assistance and culturally tailored patient support to reduce missed treatments. By contrast, the scattered SURE pockets in Figure 2c likely reflect facility-level issues best addressed through targeted quality-improvement audits, staffing support, and enhanced care coordination. As a pragmatic rule, counties in the top decile of SSRE can be prioritized for near-term resource allocation and tracked over time using the same mapping and Moran’s *I* diagnostics.

The model incorporating both spatially and aspatially structured random effects demonstrates that a substantial portion of unexplained variation is spatially structured. The inclusion of Moran eigenvector spatial filters improves the model fit by effectively capturing this latent spatial autocorrelation. While the SSRE component reveals spatial patterns, these patterns reflect unobserved spatial processes rather than directly indicating that contextual disadvantage is inherently spatial. Nonetheless, accounting for this spatial structure is essential to reduce bias and better isolate non-spatial sources of variation.

From a methodological standpoint, this study demonstrates the feasibility and value of incorporating MESF into multilevel survival modeling. The combined framework allows for more precise estimation of fixed effects while properly addressing latent spatial dependencies. The reduction in residual spatial autocorrelation confirms that MESF effectively captures structured spatial variation. Also, spatially structured random effects successfully capture spatial components that are unexplained by the independent variables as well as mitigate a potential omitted variable problem (e.g., [33]).

In conclusion, this research provides a rigorous, spatially sensitive framework for understanding ESRD survival disparities. It reveals actionable insights for health system planners and policymakers aiming to reduce inequities and improve outcomes. Future work may expand this approach by incorporating time-varying covariates, refining spatial weights matrices, and/or extending the framework to other chronic diseases and geographic landscapes. In addition, future research can extend this approach to ESRD patients in other states and other diseases.

One important takeaway from this narrative is that targeted investments and interventions should focus not just on patient care but also on the geographic structures that shape health trajectories. Addressing spatial inequalities is essential to achieving equitable ESRD treatment across Texas and beyond.

## Figures and Tables

**Table 1:** Likelihood ratio tests for the three models.

	Model 2	Model 3
Model 1	256.00 (*p*-value: 0.0000)	360.00 (*p*-value: 0.0000)
Model 2	-	102.86 (*p*-value: 0.0000)

**Table 2:** Model 1: CPH Model (Baseline).

Variable	HR	95% CI (Lower–Upper)	*p*-Value
Age 45–64	1.6555	1.5947–1.7186	0.0000 ***
Age 65–74	2.6333	2.5338–2.7367	0.0000 ***
Age 75+	4.1863	4.0263–4.3528	0.0000 ***
One transplant	0.1034	0.0958–0.1116	0.0000 ***
Two or more transplants	0.0658	0.0354–0.1223	0.0000 ***
Cardiac Death	2.1362	2.0513–2.2246	0.0000 ***
Vascular Death	2.3122	2.1965–2.4340	0.0000 ***
Disease—Cystic Kidney	0.6030	0.5459–0.6660	0.0000 ***
Disease—Glomerulonephritis	0.8146	0.7774–0.8535	0.0000 ***
Disease—Hypertension	0.9268	0.9071–0.9468	0.0000 ***
Disease—Other	1.2816	1.2443–1.3200	0.0000 ***
Disease—Other Urologic	0.8499	0.7773–0.9294	0.0004 ***
Sex (Male)	1.0046	0.9869–1.0226	0.6146
Race—American Indian/Alaska Native	0.8558	0.6790–1.0786	0.1871
Race—Asian	0.7510	0.7016–0.8040	0.0000 ***
Race—Black/African American	0.8886	0.8687–0.9090	0.0000 ***
Race—Other	0.8746	0.7673–0.9969	0.0449 **
Urbanicity Rate	0.8634	0.8163–0.9132	0.0000 ***
Percent Married	0.9983	0.9960–1.0007	0.1573
High School Graduation Rate	1.0010	0.9991–1.0029	0.3149
Poverty Rate	0.9887	0.9865–0.9909	0.0000 ***
Disability Rate	1.0090	1.0042–1.0139	0.0003 ***
Veteran Population Rate	0.9924	0.9882–0.9966	0.0004 ***
Unemployment Rate	1.0078	1.0007–1.0149	0.0307 **
AIC: 1,052,230 Log-likelihood: −526,091			

Note: *** and ** indicate the 99% and 95% significant levels; CI = Confidence Interval.

**Table 3:** Model 2: CPH Model with County-Level Random Effects.

Variable	HR	95% CI (Lower–Upper)	*p*-Value
Age 45–64	1.6580	1.5946–1.7230	0.0000 ***
Age 65–74	2.6350	2.5371–2.7384	0.0000 ***
Age 75+	4.1960	4.0310–4.3717	0.0000 ***
One transplant	0.1032	0.0956–0.1115	0.0000 ***
Two or more transplants	0.0656	0.0353–0.1219	0.0000 ***
Cardiac Death	2.1140	2.0486–2.1836	0.0000 ***
Vascular Death	2.3080	2.2157–2.4054	0.0000***
Disease—Cystic Kidney	0.6002	0.5458–0.6602	0.0000 ***
Disease—Glomerulonephritis	0.8111	0.7738–0.8509	0.0000 ***
Disease—Hypertension	0.9192	0.8988–0.9400	0.0000 ***
Disease—Other	1.2670	1.2308–1.3040	0.0000 ***
Disease—Other Urologic	0.8438	0.7645–0.9310	0.0002 ***
Sex (Male)	1.0050	0.9873–1.0231	0.5606
Race—American Indian/Alaska Native	0.8686	0.6884–1.0967	0.2338
Race—Asian	0.7396	0.6887–0.7948	0.0000 ***
Race—Black/African American	0.8662	0.8449–0.8880	0.0000 ***
Race—Other	0.8696	0.7618–0.9929	0.0368 **
Urbanicity Rate	0.9435	0.8597–1.0360	0.2005
Percent Married	0.9980	0.9931–1.0029	0.3686
High School Graduation Rate	1.0001	0.9970–1.0031	0.9654
Poverty Rate	0.9932	0.9884–0.9980	0.0059 ***
Disability Rate	1.0100	1.0029–1.0170	0.0125 **
Veteran Population Rate	1.0080	0.9999–1.0162	0.0607
Unemployment Rate	1.0030	0.9920–1.0140	0.5550
AIC: 1,051,903 Log-likelihood: −525,963 Standard deviation of the random effects: 0.1043			

Note: *** and ** indicate the 99% and 95% significant levels; CI = Confidence Interval.

**Table 4:** Model 3: Multilevel Spatial CPH Model with SSRE and SURE terms.

Variable	HR	95% CI (Lower–Upper)	*p*-Value
Age 45–64	1.6581	1.5970–1.7213	0.0000 ***
Age 65–74	2.6349	2.5354–2.7390	0.0000 ***
Age 75+	4.1956	4.0291–4.3723	0.0000 ***
One transplant	0.1033	0.0959–0.1117	0.0000 ***
Two or more transplants	0.0659	0.0355–0.1226	0.0000 ***
Cardiac Death	2.1121	2.0313–2.1942	0.0000 ***
Vascular Death	2.3038	2.1877–2.4275	0.0000 ***
Disease—Cystic Kidney	0.5995	0.5430–0.6625	0.0000 ***
Disease—Glomerulonephritis	0.8114	0.7745–0.8508	0.0000 ***
Disease—Hypertension	0.9174	0.8982–0.9378	0.0000 ***
Disease—Other	1.2648	1.2288–1.3019	0.0000 ***
Disease—Other Urologic	0.8412	0.7692–0.9197	0.0002 ***
Sex (Male)	1.0051	0.9873–1.0232	0.5737
Race—American Indian/Alaska Native	0.8718	0.6927–1.0984	0.2458
Race—Asian	0.7382	0.6920–0.7876	0.0000 ***
Race—Black/African American	0.8624	0.8420–0.8834	0.0000 ***
Race—Other	0.8708	0.7659–0.9913	0.0386 **
Urbanicity Rate	0.9361	0.8619–1.0164	0.1051
Percent Married	0.9990	0.9959–1.0020	0.6155
High School Graduation Rate	0.9993	0.9969–1.0018	0.6085
Poverty Rate	0.9967	0.9927–1.0007	0.1070
Disability Rate	1.0092	1.0016–1.0170	0.0189 **
Veteran Population Rate	1.0107	1.0036–1.0182	0.0036 ***
Unemployment Rate	1.0002	0.9900–1.0106	0.9727
AIC: 1,051,873 Log-likelihood: −525,911 Standard deviation of the random effects: 0.0519			

Note: *** and ** indicate the 99% and 95% significant levels; CI = Confidence Interval.

## Data Availability

The data reported here have been supplied by the United States Renal Data System (USRDS) [21]. The interpretation and reporting of these data are the responsibility of the authors and in no way should be seen as an official policy or interpretation of the U.S. government.
